# [μ-*N*,*N*,*N*′,*N*′-Tetra­kis(diphenyl­phosphino­meth­yl)benzene-1,4-diamine-κ^4^ *P*,*P*′:*P*′′,*P*′′′]bis­[bis­(nitrato-κ*O*)palladium(II)]

**DOI:** 10.1107/S160053680901486X

**Published:** 2009-04-30

**Authors:** Xuan-Feng Jiang, Heng-Chi Lian, Zhong Min, Xiu-Jian Wang, Jia-Huang Lin

**Affiliations:** a School of Chemistry and Chemical Engineering, Guangxi Normal University, Guilin 541004, People’s Republic of China

## Abstract

The asymmetric unit of the title complex, [Pd_2_(NO_3_)_4_(C_58_H_52_N_2_P_4_)], contains one half-mol­ecule, in which the central benzene ring is located on a crystallographic centre of inversion. The Pd atom has a distorted square-planar coordination consisting of two P and two O atoms. In the crystal structure, inter­molecular C—H⋯O inter­actions link the mol­ecules into chains, and π–π contacts between the phenyl rings [centroid–centroid distance = 3.928 (3) Å] may further stabilize the structure.

## Related literature

For related structures, see: Aucott *et al.* (2002 ▶); Ganesamoorthy *et al.* (2008 ▶); Wang *et al.* (2008 ▶). For bond-length data, see: Allen *et al.* (1987 ▶). For ring-puckering parameters, see: Cremer & Pople (1975 ▶).
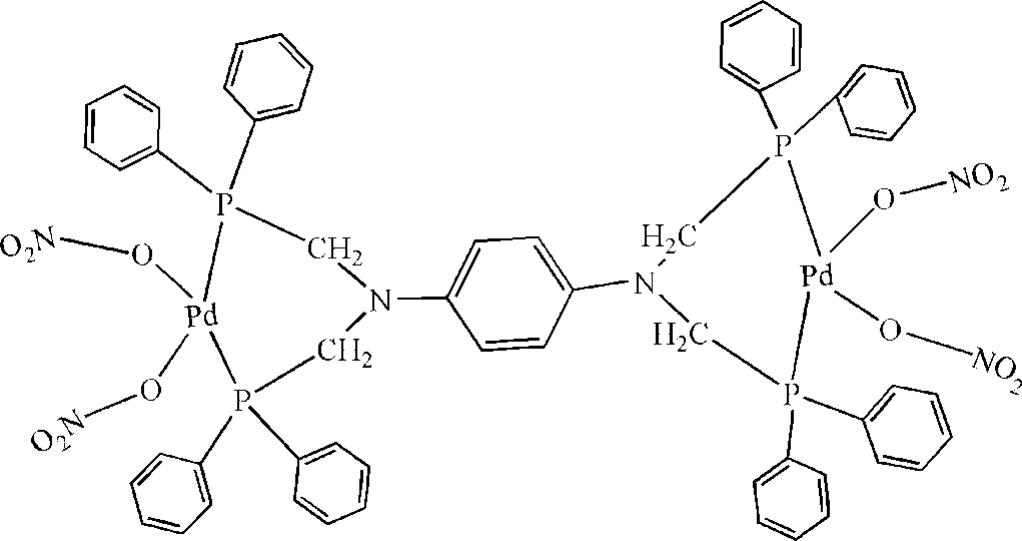


## Experimental

### 

#### Crystal data

[Pd_2_(NO_3_)_4_(C_58_H_52_N_2_P_4_)]
*M* *_r_* = 1361.74
Monoclinic, 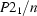

*a* = 8.0715 (2) Å
*b* = 21.3419 (7) Å
*c* = 16.0283 (5) Å
β = 92.937 (3)°
*V* = 2757.43 (14) Å^3^
*Z* = 2
Mo *K*α radiation
μ = 0.84 mm^−1^
*T* = 294 K
0.40 × 0.15 × 0.15 mm

#### Data collection

Oxford Diffraction Gemini S Untra diffractometer
Absorption correction: multi-scan (*CrysAlis RED*; Oxford Diffraction, 2007 ▶) *T*_min_ = 0.773, *T*_max_ = 0.882
13871 measured reflections
6838 independent reflections
3511 reflections with *I* > 2σ(*I*)
*R*_int_ = 0.035

#### Refinement

*R*[*F* ^2^ > 2σ(*F* ^2^)] = 0.036
*wR*(*F* ^2^) = 0.072
*S* = 0.80
6838 reflections
370 parameters
H-atom parameters constrained
Δρ_max_ = 1.04 e Å^−3^
Δρ_min_ = −0.37 e Å^−3^

### 

Data collection: *CrysAlis CCD* (Oxford Diffraction, 2007 ▶); cell refinement: *CrysAlis RED* (Oxford Diffraction, 2007 ▶); data reduction: *CrysAlis RED*; program(s) used to solve structure: *SHELXS97* (Sheldrick, 2008 ▶); program(s) used to refine structure: *SHELXL97* (Sheldrick, 2008 ▶); molecular graphics: *PLATON* (Spek, 2009 ▶); software used to prepare material for publication: *SHELXL97*.

## Supplementary Material

Crystal structure: contains datablocks I, global. DOI: 10.1107/S160053680901486X/hk2653sup1.cif

Structure factors: contains datablocks I. DOI: 10.1107/S160053680901486X/hk2653Isup2.hkl

Additional supplementary materials:  crystallographic information; 3D view; checkCIF report

## Figures and Tables

**Table:** 

Pd1—P1	2.2198 (7)
Pd1—P2	2.2087 (9)
Pd1—O3	2.144 (2)
Pd1—O6	2.1377 (17)

**Table:** 

P2—Pd1—P1	92.34 (3)
O3—Pd1—P1	89.72 (5)
O3—Pd1—P2	177.92 (5)
O3—Pd1—O6	91.77 (8)
O6—Pd1—P1	173.04 (6)
O6—Pd1—P2	86.15 (6)

**Table 2:** Hydrogen-bond geometry (Å, °)

*D*—H⋯*A*	*D*—H	H⋯*A*	*D*⋯*A*	*D*—H⋯*A*
C4—H4*B*⋯O5^i^	0.97	2.31	3.248 (3)	163

Symmetry code: (i) 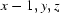.

**Fig. 1.**
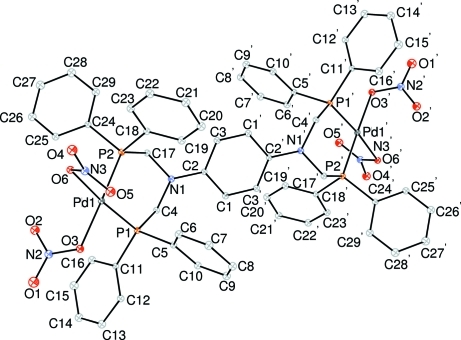
The molecular structure of the title molecule, with the atom-numbering scheme [symmetry code ('): -x, -y, -z].

## supplementary crystallographic information

### Comment

The potential applications as catalysts of palladium(II) complexes, containing phosphine ligands, had been extensively studied so far. A lot of analogs were reported (Aucott *et al.*,  2002; Ganesamoorthy *et al.*, 2008; Wang *et al.*,  2008), and some of them are catalytically active in homogeneous hydrogenations and in other organic reactions. We report herein the crystal structure of the title complex.

The asymmetric unit of the title complex contains one-half molecule (Fig. 1), in which the central benzene ring is located on a crystallographic centre of inversion. The Pd atom is in a distorted square-planar coordination by two P and two O atoms. The Pd-P and Pd-O bond lengths (Allen *et al.*, 1987) and angles (Table 1) are within normal ranges. Rings A (C5-C10), B (C11-C16), C (C18-C23) and D (C24-C29) are, of course, planar and they are oriented at dihedral angles of A/B = 64.08 (3), A/C = 17.77 (3), A/D = 78.70 (3), B/C = 64.54 (3) and B/D = 27.80 (3) °. Ring E (Pd1/P1/P2/N1/C4/C17) is not planar, having total puckering amplitude, Q_T_, of 2.670 (2) Å and twisted conformation [φ = -88.23 (3) and θ = 100.40 (3) °] (Cremer & Pople, 1975).

In the crystal structure, intermolecular C-H···O interactions (Table 2) link the molecules into chains, in which they may be effective in the stabilization of the structure.  The π–π contact between the phenyl rings, Cg2—Cg4^i^ [symmetry code: (i) 1/2 + x, 1/2 - y, z + 1/2, where Cg2 and Cg4 are centroids of the rings B (C11-C16) and D (C24-C29), respectively] may further stabilize the structure, with centroid-centroid distance of 3.928 (3) Å.

### Experimental

For the preparation of the title complex, PdCl_2_(0.0350 g, 0.2 mmol) in CH_3_CN (10 ml) was stirred at 348-353 K for 4 h, until the solid palladium salt was dissolved completely to give a yellow solution. Then, the liquor was cooled to about 277 K for a few hours, and the yellow precipitate, Pd(CH_3_CN)_2_Cl_2_, was collected by filtration. Then, Pd(CH_3_CN)_2_Cl_2_ (0.0265 g, 0.1 mmol) in CH_3_CN (5 ml) was stirred at room temperature for 20 min, and AgNO_3_ (0.0345 g, 0.2 mmol) in DMF (5 ml) was added dropwise with stirring. A pale precipitate of AgCl formed and the solution was filtered, The phosphine ligand, (L), (0.0450 g, 0.05 mmol) was added until the color of solution turned to red, and then the stirring is continued for 2 h. After filtration, diffusion of diethyl ether at room temperature into the solution the yellow crystals of the title complex, [Pd_2_(L)(NO_3_)_4_], were obtained.

### Refinement

H atoms were positioned geometrically, with C-H = 0.93 and 0.97 Å for aromatic and methylene H, respectively, and constrained to ride on their parent atoms, with U_iso_(H) = 1.2U_eq_(C).

### Crystal data

**Table:**

[Pd_2_(NO_3_)_4_(C_58_H_52_N_2_P_4_)]	*F*(000) = 1380
*M**_r_* = 1361.74	*D*_x_ = 1.640 Mg m^−^^3^
Monoclinic, *P*2_1_/*n*	Mo *K*α radiation, λ = 0.71073 Å
Hall symbol: -P 2yn	Cell parameters from 4249 reflections
*a* = 8.0715 (2) Å	θ = 11.3–28.6°
*b* = 21.3419 (7) Å	µ = 0.84 mm^−^^1^
*c* = 16.0283 (5) Å	*T* = 294 K
β = 92.937 (3)°	Block, yellow
*V* = 2757.43 (14) Å^3^	0.4 × 0.15 × 0.15 mm
*Z* = 2	

### Data collection

**Table:**

Gemini S Untra diffractometer	6838 independent reflections
Radiation source: Enhance (Mo) X-ray Source	3511 reflections with *I* > 2σ(*I*)
graphite	*R*_int_ = 0.035
Detector resolution: 16.0855 pixels mm^-1^	θ_max_ = 30.2°, θ_min_ = 2.8°
ω scans	*h* = −11→9
Absorption correction: multi-scan (*CrysAlis RED*; Oxford Diffraction, 2007)	*k* = −28→27
*T*_min_ = 0.773, *T*_max_ = 0.882	*l* = −22→21
13871 measured reflections	

### Refinement

**Table:**

Refinement on *F*^2^	Primary atom site location: structure-invariant direct methods
Least-squares matrix: full	Secondary atom site location: difference Fourier map
*R*[*F*^2^ > 2σ(*F*^2^)] = 0.036	Hydrogen site location: inferred from neighbouring sites
*wR*(*F*^2^) = 0.072	H-atom parameters constrained
*S* = 0.80	*w* = 1/[σ^2^(*F*_o_^2^) + (0.0313*P*)^2^] where *P* = (*F*_o_^2^ + 2*F*_c_^2^)/3
6838 reflections	(Δ/σ)_max_ = 0.004
370 parameters	Δρ_max_ = 1.04 e Å^−^^3^
0 restraints	Δρ_min_ = −0.37 e Å^−^^3^

### Special details

**Table:**

Geometry. All e.s.d.'s (except the e.s.d. in the dihedral angle between two l.s. planes) are estimated using the full covariance matrix. The cell e.s.d.'s are taken into account individually in the estimation of e.s.d.'s in distances, angles and torsion angles; correlations between e.s.d.'s in cell parameters are only used when they are defined by crystal symmetry. An approximate (isotropic) treatment of cell e.s.d.'s is used for estimating e.s.d.'s involving l.s. planes.
Refinement. Refinement of *F*^2^ against ALL reflections. The weighted *R*-factor *wR* and goodness of fit *S* are based on *F*^2^, conventional *R*-factors *R* are based on *F*, with *F* set to zero for negative *F*^2^. The threshold expression of *F*^2^ > σ(*F*^2^) is used only for calculating *R*-factors(gt) *etc*. and is not relevant to the choice of reflections for refinement. *R*-factors based on *F*^2^ are statistically about twice as large as those based on *F*, and *R*- factors based on ALL data will be even larger.

### Fractional atomic coordinates and isotropic or equivalent isotropic displacement parameters (Å^2^)

**Table:**

	*x*	*y*	*z*	*U*_iso_*/*U*_eq_	
Pd1	1.39486 (3)	1.789182 (12)	0.214196 (13)	0.02504 (8)	
P1	1.22599 (9)	1.78487 (4)	0.10059 (4)	0.02366 (18)	
P2	1.30017 (9)	1.88209 (4)	0.24940 (5)	0.02471 (19)	
O1	1.4811 (3)	1.60188 (12)	0.22173 (15)	0.0613 (8)	
O2	1.3721 (3)	1.67330 (12)	0.29761 (14)	0.0461 (6)	
O3	1.4938 (2)	1.69934 (10)	0.18353 (12)	0.0311 (5)	
O4	1.8332 (3)	1.83114 (12)	0.33338 (14)	0.0518 (7)	
O5	1.7301 (3)	1.81444 (13)	0.20792 (14)	0.0486 (7)	
O6	1.5755 (2)	1.80091 (10)	0.31524 (11)	0.0298 (5)	
N1	1.0910 (3)	1.90013 (12)	0.11125 (14)	0.0271 (6)	
N2	1.4459 (3)	1.65651 (15)	0.23480 (18)	0.0391 (7)	
N3	1.7185 (3)	1.81587 (13)	0.28399 (18)	0.0372 (7)	
C1	0.9830 (3)	1.93941 (14)	−0.02623 (17)	0.0299 (8)	
H1A	0.9729	1.8985	−0.0457	0.036*	
C2	1.0433 (3)	1.94993 (14)	0.05573 (16)	0.0240 (7)	
C3	1.0617 (3)	2.01198 (14)	0.07878 (18)	0.0286 (8)	
H3A	1.1061	2.0212	0.1320	0.034*	
C4	1.0437 (3)	1.83578 (14)	0.09201 (17)	0.0264 (7)	
H4A	0.9936	1.8335	0.0358	0.032*	
H4B	0.9624	1.8217	0.1304	0.032*	
C5	1.3384 (3)	1.80561 (14)	0.00986 (17)	0.0264 (7)	
C6	1.5000 (4)	1.82739 (15)	0.0189 (2)	0.0352 (8)	
H6A	1.5527	1.8304	0.0717	0.042*	
C7	1.5828 (4)	1.84462 (17)	−0.0511 (2)	0.0417 (9)	
H7A	1.6916	1.8589	−0.0451	0.050*	
C8	1.5067 (4)	1.84081 (16)	−0.1282 (2)	0.0390 (8)	
H8A	1.5637	1.8523	−0.1748	0.047*	
C9	1.3461 (4)	1.82008 (16)	−0.1380 (2)	0.0378 (9)	
H9A	1.2945	1.8180	−0.1911	0.045*	
C10	1.2609 (4)	1.80238 (15)	−0.06993 (18)	0.0321 (8)	
H10A	1.1520	1.7883	−0.0769	0.039*	
C11	1.1447 (3)	1.70636 (15)	0.08770 (17)	0.0249 (7)	
C12	1.2026 (4)	1.66574 (15)	0.02931 (19)	0.0305 (8)	
H12A	1.2794	1.6793	−0.0081	0.037*	
C13	1.1461 (5)	1.60480 (16)	0.0265 (2)	0.0422 (9)	
H13A	1.1848	1.5774	−0.0132	0.051*	
C14	1.0363 (4)	1.58451 (16)	0.0802 (2)	0.0438 (9)	
H14A	0.9999	1.5432	0.0776	0.053*	
C15	0.9767 (5)	1.62465 (18)	0.1396 (2)	0.0497 (10)	
H15A	0.8998	1.6104	0.1765	0.060*	
C16	1.0314 (4)	1.68549 (16)	0.1437 (2)	0.0387 (9)	
H16A	0.9928	1.7126	0.1839	0.046*	
C17	1.1050 (3)	1.91098 (15)	0.20009 (16)	0.0267 (7)	
H17A	1.0131	1.8906	0.2258	0.032*	
H17B	1.0963	1.9556	0.2105	0.032*	
C18	1.4595 (4)	1.93885 (15)	0.23045 (18)	0.0279 (7)	
C19	1.4731 (4)	1.96391 (15)	0.15109 (19)	0.0353 (8)	
H19A	1.3939	1.9545	0.1088	0.042*	
C20	1.6040 (4)	2.00260 (17)	0.1352 (2)	0.0411 (9)	
H20A	1.6123	2.0196	0.0822	0.049*	
C21	1.7229 (4)	2.01633 (16)	0.1973 (2)	0.0404 (9)	
H21A	1.8105	2.0429	0.1863	0.048*	
C22	1.7118 (4)	1.99062 (17)	0.2755 (2)	0.0398 (9)	
H22A	1.7926	1.9995	0.3172	0.048*	
C23	1.5816 (4)	1.95192 (15)	0.29200 (19)	0.0348 (8)	
H23A	1.5752	1.9344	0.3448	0.042*	
C24	1.2525 (3)	1.88447 (15)	0.35784 (17)	0.0258 (7)	
C25	1.2340 (4)	1.83006 (16)	0.40314 (18)	0.0320 (8)	
H25A	1.2617	1.7917	0.3802	0.038*	
C26	1.1751 (4)	1.83253 (18)	0.4817 (2)	0.0410 (9)	
H26A	1.1623	1.7957	0.5117	0.049*	
C27	1.1346 (4)	1.8891 (2)	0.5166 (2)	0.0490 (10)	
H27A	1.0932	1.8903	0.5696	0.059*	
C28	1.1551 (4)	1.9432 (2)	0.4734 (2)	0.0479 (10)	
H28A	1.1301	1.9814	0.4976	0.058*	
C29	1.2133 (4)	1.94164 (17)	0.39349 (19)	0.0374 (8)	
H29A	1.2260	1.9787	0.3639	0.045*	

### Atomic displacement parameters (Å^2^)

**Table:**

	*U*^11^	*U*^22^	*U*^33^	*U*^12^	*U*^13^	*U*^23^
Pd1	0.02445 (13)	0.02725 (13)	0.02345 (13)	0.00320 (13)	0.00145 (9)	0.00167 (13)
P1	0.0258 (4)	0.0238 (4)	0.0215 (4)	0.0005 (4)	0.0019 (3)	0.0011 (4)
P2	0.0240 (4)	0.0260 (5)	0.0242 (4)	0.0002 (4)	0.0017 (3)	0.0016 (4)
O1	0.092 (2)	0.0341 (16)	0.0588 (17)	0.0263 (16)	0.0099 (14)	0.0110 (14)
O2	0.0473 (15)	0.0508 (17)	0.0411 (15)	0.0053 (13)	0.0123 (12)	0.0066 (13)
O3	0.0336 (12)	0.0306 (13)	0.0294 (12)	0.0073 (11)	0.0068 (9)	0.0021 (11)
O4	0.0329 (14)	0.0729 (19)	0.0481 (15)	−0.0094 (14)	−0.0126 (11)	0.0088 (14)
O5	0.0395 (14)	0.078 (2)	0.0293 (13)	−0.0059 (14)	0.0083 (10)	0.0091 (13)
O6	0.0195 (11)	0.0434 (15)	0.0266 (11)	0.0012 (11)	0.0019 (8)	0.0020 (10)
N1	0.0311 (15)	0.0222 (14)	0.0275 (14)	0.0011 (13)	−0.0017 (11)	−0.0015 (12)
N2	0.0383 (17)	0.044 (2)	0.0356 (18)	0.0165 (16)	0.0058 (13)	0.0134 (17)
N3	0.0301 (17)	0.0392 (17)	0.0421 (18)	0.0000 (14)	−0.0012 (14)	0.0075 (15)
C1	0.0334 (18)	0.0218 (17)	0.0339 (18)	0.0022 (15)	−0.0028 (14)	−0.0037 (15)
C2	0.0243 (17)	0.0253 (17)	0.0226 (17)	0.0047 (15)	0.0042 (13)	0.0035 (15)
C3	0.0331 (18)	0.0288 (19)	0.0235 (16)	0.0015 (16)	−0.0044 (13)	−0.0022 (15)
C4	0.0241 (17)	0.0296 (18)	0.0258 (17)	0.0001 (15)	0.0036 (13)	0.0042 (15)
C5	0.0266 (17)	0.0259 (18)	0.0268 (17)	0.0001 (14)	0.0038 (13)	0.0016 (14)
C6	0.035 (2)	0.035 (2)	0.0363 (19)	0.0006 (18)	0.0026 (15)	0.0065 (17)
C7	0.035 (2)	0.050 (2)	0.040 (2)	−0.0088 (19)	0.0065 (16)	0.0088 (19)
C8	0.042 (2)	0.044 (2)	0.0331 (19)	−0.0032 (19)	0.0169 (15)	0.0040 (17)
C9	0.048 (2)	0.034 (2)	0.0313 (19)	0.0001 (19)	0.0014 (16)	0.0023 (17)
C10	0.0289 (17)	0.036 (2)	0.0320 (18)	−0.0045 (16)	0.0041 (14)	0.0015 (16)
C11	0.0276 (17)	0.0246 (17)	0.0222 (15)	0.0014 (16)	−0.0017 (13)	0.0026 (16)
C12	0.0293 (18)	0.0280 (19)	0.0346 (18)	0.0013 (16)	0.0043 (14)	0.0048 (17)
C13	0.063 (3)	0.024 (2)	0.039 (2)	0.0072 (19)	0.0000 (18)	−0.0046 (18)
C14	0.064 (3)	0.0213 (19)	0.045 (2)	−0.0073 (19)	−0.0067 (19)	0.0044 (18)
C15	0.066 (3)	0.043 (2)	0.042 (2)	−0.018 (2)	0.0138 (18)	0.008 (2)
C16	0.049 (2)	0.032 (2)	0.035 (2)	−0.0066 (18)	0.0085 (16)	0.0010 (17)
C17	0.0216 (16)	0.0277 (18)	0.0310 (18)	0.0059 (15)	0.0035 (13)	0.0050 (15)
C18	0.0253 (17)	0.0257 (18)	0.0328 (18)	0.0046 (15)	0.0016 (13)	−0.0034 (15)
C19	0.0320 (19)	0.033 (2)	0.041 (2)	−0.0028 (17)	0.0057 (15)	0.0012 (17)
C20	0.038 (2)	0.041 (2)	0.045 (2)	0.0015 (19)	0.0106 (16)	0.0052 (18)
C21	0.037 (2)	0.029 (2)	0.056 (2)	−0.0055 (17)	0.0153 (17)	−0.0056 (19)
C22	0.0274 (19)	0.039 (2)	0.054 (2)	−0.0034 (17)	0.0044 (15)	−0.0153 (19)
C23	0.040 (2)	0.034 (2)	0.0301 (18)	0.0011 (17)	0.0053 (15)	−0.0023 (17)
C24	0.0182 (16)	0.0330 (19)	0.0260 (16)	−0.0040 (15)	0.0005 (12)	0.0046 (16)
C25	0.0304 (18)	0.039 (2)	0.0266 (18)	−0.0057 (17)	0.0020 (14)	−0.0024 (17)
C26	0.042 (2)	0.048 (3)	0.032 (2)	−0.017 (2)	0.0028 (16)	0.0041 (19)
C27	0.044 (2)	0.072 (3)	0.032 (2)	−0.018 (2)	0.0144 (16)	−0.014 (2)
C28	0.044 (2)	0.055 (3)	0.046 (2)	−0.002 (2)	0.0145 (17)	−0.013 (2)
C29	0.038 (2)	0.039 (2)	0.036 (2)	−0.0014 (18)	0.0014 (15)	0.0011 (18)

### Geometric parameters (Å, °)

**Table:**

Pd1—P1	2.2198 (7)	C10—H10A	0.9300
Pd1—P2	2.2087 (9)	C11—C12	1.375 (4)
Pd1—O3	2.144 (2)	C11—C16	1.388 (4)
Pd1—O6	2.1377 (17)	C12—C13	1.378 (4)
P1—C4	1.829 (3)	C12—H12A	0.9300
P1—C5	1.808 (3)	C13—C14	1.338 (5)
P1—C11	1.807 (3)	C13—H13A	0.9300
P2—C17	1.833 (3)	C14—C15	1.385 (5)
P2—C18	1.803 (3)	C14—H14A	0.9300
P2—C24	1.800 (3)	C15—C16	1.372 (5)
O1—N2	1.221 (4)	C15—H15A	0.9300
O2—N2	1.248 (3)	C16—H16A	0.9300
O3—N2	1.301 (3)	C17—H17A	0.9700
O4—N3	1.231 (3)	C17—H17B	0.9700
O5—N3	1.228 (3)	C18—C19	1.389 (4)
O6—N3	1.320 (3)	C18—C23	1.387 (4)
N1—C2	1.426 (3)	C19—C20	1.375 (4)
N1—C4	1.454 (4)	C19—H19A	0.9300
N1—C17	1.441 (3)	C20—C21	1.378 (4)
C1—C2	1.396 (4)	C20—H20A	0.9300
C1—C3^i^	1.373 (4)	C21—C22	1.376 (4)
C1—H1A	0.9300	C21—H21A	0.9300
C2—C3	1.381 (4)	C22—C23	1.373 (4)
C3—C1^i^	1.373 (4)	C22—H22A	0.9300
C3—H3A	0.9300	C23—H23A	0.9300
C4—H4A	0.9700	C24—C25	1.381 (4)
C4—H4B	0.9700	C24—C29	1.391 (4)
C5—C6	1.386 (4)	C25—C26	1.370 (4)
C5—C10	1.396 (4)	C25—H25A	0.9300
C6—C7	1.384 (4)	C26—C27	1.376 (5)
C6—H6A	0.9300	C26—H26A	0.9300
C7—C8	1.354 (4)	C27—C28	1.360 (5)
C7—H7A	0.9300	C27—H27A	0.9300
C8—C9	1.371 (4)	C28—C29	1.387 (4)
C8—H8A	0.9300	C28—H28A	0.9300
C9—C10	1.372 (4)	C29—H29A	0.9300
C9—H9A	0.9300		
			
P2—Pd1—P1	92.34 (3)	C9—C10—H10A	120.2
O3—Pd1—P1	89.72 (5)	C12—C11—P1	121.9 (2)
O3—Pd1—P2	177.92 (5)	C12—C11—C16	119.8 (3)
O3—Pd1—O6	91.77 (8)	C16—C11—P1	118.1 (2)
O6—Pd1—P1	173.04 (6)	C11—C12—C13	119.6 (3)
O6—Pd1—P2	86.15 (6)	C11—C12—H12A	120.2
C4—P1—Pd1	119.67 (10)	C13—C12—H12A	120.2
C5—P1—Pd1	109.72 (9)	C12—C13—H13A	119.5
C5—P1—C4	103.24 (13)	C14—C13—C12	120.9 (3)
C11—P1—Pd1	109.65 (9)	C14—C13—H13A	119.5
C11—P1—C4	104.84 (13)	C13—C14—C15	120.4 (3)
C11—P1—C5	109.21 (14)	C13—C14—H14A	119.8
C17—P2—Pd1	119.53 (10)	C15—C14—H14A	119.8
C18—P2—Pd1	107.51 (10)	C14—C15—H15A	120.1
C18—P2—C17	107.81 (14)	C16—C15—C14	119.8 (3)
C24—P2—Pd1	111.46 (11)	C16—C15—H15A	120.1
C24—P2—C17	100.67 (13)	C11—C16—H16A	120.2
C24—P2—C18	109.47 (14)	C15—C16—C11	119.6 (3)
N2—O3—Pd1	110.94 (18)	C15—C16—H16A	120.2
N3—O6—Pd1	108.45 (16)	P2—C17—H17A	108.9
C2—N1—C4	120.8 (2)	P2—C17—H17B	108.9
C2—N1—C17	120.1 (2)	N1—C17—P2	113.2 (2)
C17—N1—C4	111.6 (2)	N1—C17—H17A	108.9
O1—N2—O2	122.5 (3)	N1—C17—H17B	108.9
O1—N2—O3	118.9 (3)	H17A—C17—H17B	107.7
O2—N2—O3	118.5 (3)	C19—C18—P2	120.2 (2)
O4—N3—O5	123.5 (3)	C23—C18—P2	120.1 (2)
O4—N3—O6	117.6 (3)	C23—C18—C19	119.1 (3)
O5—N3—O6	118.9 (2)	C18—C19—H19A	120.0
C2—C1—H1A	119.2	C20—C19—C18	119.9 (3)
C3^i^—C1—C2	121.6 (3)	C20—C19—H19A	120.0
C3^i^—C1—H1A	119.2	C19—C20—C21	120.5 (3)
C1—C2—N1	122.5 (3)	C19—C20—H20A	119.8
C3—C2—C1	115.7 (3)	C21—C20—H20A	119.8
C3—C2—N1	121.7 (2)	C20—C21—H21A	120.1
C1^i^—C3—C2	122.6 (3)	C22—C21—C20	119.8 (3)
C1^i^—C3—H3A	118.7	C22—C21—H21A	120.1
C2—C3—H3A	118.7	C21—C22—H22A	119.9
P1—C4—H4A	109.6	C23—C22—C21	120.1 (3)
P1—C4—H4B	109.6	C23—C22—H22A	119.9
N1—C4—P1	110.08 (19)	C18—C23—H23A	119.8
N1—C4—H4A	109.6	C22—C23—C18	120.5 (3)
N1—C4—H4B	109.6	C22—C23—H23A	119.8
H4A—C4—H4B	108.2	C25—C24—C29	119.3 (3)
C6—C5—P1	120.5 (2)	C25—C24—P2	121.2 (2)
C6—C5—C10	119.2 (3)	C29—C24—P2	119.1 (2)
C10—C5—P1	120.3 (2)	C24—C25—H25A	119.9
C5—C6—H6A	120.1	C26—C25—C24	120.2 (3)
C7—C6—C5	119.7 (3)	C26—C25—H25A	119.9
C7—C6—H6A	120.1	C25—C26—C27	120.5 (4)
C6—C7—H7A	119.7	C25—C26—H26A	119.8
C8—C7—C6	120.5 (3)	C27—C26—H26A	119.8
C8—C7—H7A	119.7	C26—C27—H27A	120.0
C7—C8—C9	120.4 (3)	C28—C27—C26	120.0 (3)
C7—C8—H8A	119.8	C28—C27—H27A	120.0
C9—C8—H8A	119.8	C27—C28—C29	120.4 (4)
C8—C9—C10	120.5 (3)	C27—C28—H28A	119.8
C8—C9—H9A	119.7	C29—C28—H28A	119.8
C10—C9—H9A	119.7	C24—C29—H29A	120.2
C5—C10—H10A	120.2	C28—C29—C24	119.7 (3)
C9—C10—C5	119.7 (3)	C28—C29—H29A	120.2

Symmetry codes: (i) −*x*+2, −*y*+4, −*z*.

### Hydrogen-bond geometry (Å, °)

**Table:**

*D*—H···*A*	*D*—H	H···*A*	*D*···*A*	*D*—H···*A*
C4—H4B···O5^ii^	0.97	2.31	3.248 (3)	163

Symmetry codes: (ii) *x*−1, *y*, *z*.
